# Comprehensive prognostic analysis in breast cancer integrating clinical, tumoral, micro-environmental and immunohistochemical criteria

**DOI:** 10.1186/s40064-015-1297-8

**Published:** 2015-09-21

**Authors:** Isabelle de Mascarel, Marc Debled, Véronique Brouste, Louis Mauriac, Ghislaine Sierankowski, Valérie Velasco, Sabrina Croce, Frédéric Chibon, Jêrome Boudeau, Anne Debant, Gaëtan MacGrogan

**Affiliations:** Department of BioPathology, Institut Bergonié, 229, cours de l’Argonne, 33076 Bordeaux, France; Department of Medical Oncology, Institut Bergonié, 229 cours de l’Argonne, 33076 Bordeaux, France; Clinical and Epidemiological Research Unit, Institut Bergonié, 229 cours de l’Argonne, 33076 Bordeaux, France; Institut National de la Santé et de la Recherche Médicale (INSERM) U916, Institut Bergonié, 229 cours de l’Argonne, 33076 Bordeaux, France; Centre de Recherche en Biochimie Macromoléculaire, CRBM-CNRS UMR 5237, Universités Montpellier I et II, Montpellier, France

**Keywords:** BCL2, Breast neoplasms, Estrogen receptor, Bcl2, Claudin-low, Rho GEF Trio

## Abstract

Significant morphological, clinical and biological prognostic factors vary according to molecular subtypes of breast tumors, yet comprehensive analysis of such factors linked to survival in each group is lacking. Clinicopathological and micro-environmental criteria, estrogen (ER), progesterone (PR) receptors, HER2, Ki67, basal markers, CD24, CD44, ALDH1, BCL2, E-Cadherin and Trio were assessed in 1070 primary operable breast cancers from a single center according to five main molecular subtypes and associations with distant metastasis-free survival (DMFS) were examined. There were 682 (64 %) luminal A (LA), 166 (16 %) Luminal B HER2 negative (LBH−), 47 (4 %) Luminal B HER2 positive (LBH+), 108 (10 %) triple negative (TN) and 67 (6 %) HER2-enriched tumors (H2+). Median follow-up was 13.7 years. At 5 years, DMFS in LA (90 %) was better than in LBH− (80.9 %), hazard ratio (HR) = 2.22 [1.44–3.43] *P* < 0.001; LBH+ (74.5 %), HR = 3.14 [1.69–5.84] *P* < 0.001, TN (71.5 %) HR = 3.63 [2.34–5.63], *P* < 0.001; and H2+ (65.2 %), HR = 4.69 [2.90–7.59], *P* < 0.001. In multivariable analysis, factors associated with shorter DMFS varied according to molecular subtype, with tumor size being associated with shorter DMFS in the LBH−, LBH+ and TN groups and the Rho GEF Trio and BCL2 phenotypes in TN tumors only. These findings help to define new clinicophenotypic models and to identify new therapeutic strategies in the specific molecular subgroups.

**Electronic supplementary material:**

The online version of this article (doi:10.1186/s40064-015-1297-8) contains supplementary material, which is available to authorized users.

## Background

The 2012 WHO classification based solely on the morphological features of breast tumors has limited utility for clinical and therapeutic management (Lakhani et al. 2012). Perou et al. classified breast cancers according to molecular subtypes: luminal A (LA), luminal B (LB), triple-negative (TN), HER2-enriched (H2+) and normal breast-like tumors (Perou et al. 2000). However, about half of the HER2 overexpressing tumors are ER-positive and fall into the LB subtype, the normal breast-like group seems to be an artifact, and ER-negative tumors encompass at least three other molecular subgroups: the molecular apocrine (MA) breast tumor group, the interferon-rich subgroup and the claudin-low subtype.

While several studies have demonstrated the prognostic value of the intrinsic classification (Sorlie et al. 2001) or of morphological, clinical or biological prognostic factors according to molecular type (Maiorano et al. 2010; Pages et al. 2010; Rajput et al. 2008; Tan et al. 2011), most studies have presented only one type of criteria, for example micro-environmental (Maiorano et al. 2010; Pages et al. 2010; Rajput et al. 2008; Tan et al. 2011), biological (Lane et al. 2008) or immunohistochemical (Blows et al. 2010; Cheang et al. 2008, 2009). Furthermore, for the immunohistochemical criteria, only certain antibodies in certain molecular groups are presented (Charpin et al. 2009; Dawson et al. 2010; Kim et al. 2011; Morrison et al. 2008), or only for one molecular subtype (Cheang et al. 2009).

Here, we report a comprehensive prognostic analysis that integrates clinicopathological criteria, micro-environmental parameters and selected immunohistochemical markers focusing on the five main molecular groups of breast cancers Luminal A (LA), Luminal B HER2-negative (LBH−), Luminal B HER2-positive (LBH+), Triple negative (TN) and HER2-enriched (H2+) as defined by Goldhirsch et al. (2013) and Maisonneuve et al. (2014). For each molecular group, we describe parameters, identify particular sub-phenotypes [Basal (CK5/6 and/or vimentin and/or EGFR positive), Claudin-low (CD24−/CD44+ and/or ALDH1 positive), E-Cadherin, Trio and BCL2], and report prognostic factors of distant metastatic-free survival (DMFS).

## Methods

### Patient selection

Between 1989 and 1993, 1634 patients with primary operable invasive breast carcinoma were operated on and monitored at our institution. Tissue microarray (TMA) blocks were built for immunohistochemical analyses and 1070 cases were available for our study [969 invasive ductal carcinomas, not otherwise specified (IDC-NOS) and 101 invasive lobular carcinomas (ILC)]. Median follow-up was 13.7 years [95 %CI (3.6–14)]. Patients were operated on, either by a modified radical mastectomy (260 cases) or local tumor resection (810 cases), with axillary node dissection in 1037 cases (98 %). Post-operative breast irradiation was performed in 835 cases. Adjuvant therapy with chemotherapy and/or hormone therapy was decided according to nodal status and hormone receptor determination results. Chemotherapy regimens included cyclophosphamide, methotextrate, 5-fluorouracil or epirubicin, vincristine, methotextrate; or mitomycin-C, thiotepa, vindesin. All patients were followed in a single large referral center. Institutional review board approval was obtained for this retrospective study in accordance with national laws.

### Classical and micro-environmental pathological parameters

Classical and micro-environmental parameters that had been prospectively entered in a breast cancer data base were available for analysis. The largest macroscopic and/or microscopic tumor size, and only definite peritumoral invasion were taken into account. Mitotic index was calculated as a function of the number of observed mitoses in 10 high-power fields (X400): scores 1, 2 and 3 were respectively associated with <5, 5–15 and >15 mitoses. The median number of lymph nodes examined per case was 14 (range 2–35). Inflammation, fibrosis, elastosis or necrosis, evaluated on whole tissue sections ≥50 % were considered as marked.

### TMA and immuno-histochemical assay

Eleven IDC-NOS and six ILC tissue microarray blocks were constructed. For each case, four (for IDC-NOS) and six (for ILC) 0.6 mm cores were performed. Every TMA block from the 1070 patients was re-cut into 4 μm sections mounted on SuperFrost^®^ slides. Full details on immunohistochemisty processes are detailed in Additional file 1: Annex A. We also tested Trio, a complex protein activating Rho-GTPases which plays a role in cell adhesion, motility and invasion through the control of actin cytoskeleton remodeling.

### Interpretation of immuno-histochemical assays

Tumors were considered as positive for estrogen (ER) and progesterone (PR) receptors when ≥1 % of cells showed expression and for HER2 if the Her2 immunostain showed 3+ staining intensity. For 2+ scores (n = 38), whole tissue sections were reviewed and among them, 17 tumors were re-evaluated as 3+ and 21 as 2+. FISH analysis was not performed in these 21 cases (1.8 % of the study population) as it is not interpretable in Hollande-fixed tumors. In these 21 cases we inferred their HER2 status based on our previous studies: tumors with more than 60 % of 2+ positive tumor cells were considered as HER2 positive (Chibon et al. 2009). Two cut-offs of 14 and 19 % were chosen for Ki67 classifications (Maisonneuve et al. 2014). Cases were categorized as basal immunophenotype when at least one out of the three markers (CK5/6, EGFR, and vimentin) was positive. Expression of other markers was considered as positive when 1 % or more of cells expressed them.

### Definition of the phenotypic groups

The five main phenotypic groups according to the Saint Gallen (Goldhirsch et al. 2013) classification modified by Maisonneuve et al. (2014) are as follows:

LA: ER positive, HER2 negative and Ki67 < 14 % *or* 14 % ≤ Ki67 ≤ 19 % and PR ≥ 20 %;

LBH−: ER positive, HER2 negative and Ki67 > 19 % *or* 14 % ≤ Ki67 ≤ 19 % and PR < 20 %; LBH+: ER positive and HER2 positive, TN: ER and PR negative and HER2 negative, and H2+: ER and PR negative and HER2 positive.

Within the five main molecular groups, we also sought to describe tumors by other sub phenotypes: basal (CK5/6 and/or vimentin and/or EGFR positive), Claudin-low (CD24−/CD44+ and/or ALDH1 positive), E-Cadherin, Trio and BCL2 phenotypes. Thus, the TN group was further divided into basal-like (ER/PR/HER2 negative, CK5/6 and/or vimentin and/or EGFR positive (Nielsen et al. 2004)) or non-basal-like (ER/PR/HER2 negative, CK5/6, vimentin and EGFR negative) for descriptive purposes.

### Statistical considerations

We compared the χ^2^ values of data from the five molecular subgroups. Comparisons were made in the ER/PR positive groups (LA vs. LBH−, LA vs LBH+, LBH− vs LBH+), in the ER/PR negative groups (TN vs. H2+) and the HER2 positive groups (LBH+ vs H2+), and overall between the five main phenotypic groups. Quantitative data was transformed into categorical variables and when cases were not sufficient for inter-category comparisons, category regroupings were made based on clinical justifications. Median follow-up was calculated by the reverse Kaplan–Meier method where deaths are censored and survival was recorded as the time between treatment and the last news or death. All distant metastatic events were taken into account for DMFS analysis and patients with no metastases (alive or deceased) were censored at the date of last news or death. Risk proportionality was tested with the residuals test. As DMFS risk varies over time, two Cox models were fitted over different time periods: the first 5 years then after 5 years, corresponding approximately to the median time to first event (Bellera et al. 2010). Five-year DMFS was calculated for all patients, and 10-year DMFS was calculated only for patients with no events in the first time period, and thus considered still at risk.

Univariate and multivariate analyses were calculated with hazard ratios (HR) and a Cox model for DMFS over the two time periods with no risk proportionality violations. Only variables significant at 0.05 in the univariate were maintained in the multivariate models which used a stepwise ascending maximum likelihood method.

## Results

Among the 1070 breast tumors, 682 (64 %) were Luminal A (LA), 166 (16 %) were Luminal B HER2 negative (LBH−), 47 (4 %) were Luminal B HER2 positive (LBH+), 108 (10 %) were triple negative (TN) and 67 (6 %) were HER2-enriched tumors (H2+). Among the TN tumors, there were 88 (8.5 %) basal-like and 16 (1.5 %) non-basal-like phenotypes.

### Clinicopathological and micro-environmental features across molecular groups

Clinical and morphological features differed significantly in the different molecular groups (Additional file 2: Tables S1a–e). Other than ki-67 and PR levels, major differences between LA and LBH− were age, tumor size, mitotic count, SBR grade, nodal involvement, vascular invasion, elastosis, necrosis and inflammation. The same differences were observed between LA and LBH+; nodal status and fibrosis were not significantly different in these two molecular groups. LBH+ patients were younger and less often node-positive compared to the LBH− group. Patients in the H2+ group were older compared to LBH+ patients, their tumors were more often grade 3, with a higher mitotic count, more inflammation and more necrosis. Clinicopathological features including the micro-environment were similar in TN and H2+ groups (except, nodal status). Comparisons across the five groups revealed several overall differences, for example, advanced age of LA patients in general and tumor necrosis more frequent in TN and HER2-enriched.

### Particular phenotypes within molecular groups

Immunohistochemical parameters within the molecular groups identifying the five specific phenotypes are presented in Table 1. Overall, 201 tumors showed a basal phenotype (CK5/6 and/or vimentin and/or EGFR positive) (19 %) including 71 (35 %) HR positive and 130 (65 %) HR negative tumors. The CD24−/CD44+ Claudin-low phenotype (CD44+/CD24−/low or ALDH1 positive) was infrequent in the LBH+ (8.5 %) and H2+ (19 %) tumors and more frequent in TN (41 %) tumors. The CD24+/CD44− immunophenotype was more frequent in LBH− (27 %) and LBH+ (38 %) than in LA (14 %) tumors. There was no difference in expression of Trio according to the molecular groups. BCL2 expressed significantly more frequently in HR positive than in HR negative tumors.

**Table 1:** Immunohistochemical factors and molecular groups

	LA (n = 682)	LBH− (N = 166)	LBH+ (N = 47)	H2+ (n = 67)	TN (n = 108)
Estrogen receptor					
0 %				67 (100.0)	108 (100.0)
1–9 %	14 (2.1)	4 (2.4 %)	5 (10.6 %)		
≥10 %	668 (97.9)	162 (97.6 %)	42 (89.4 %)		
Progesterone receptor					
0 %	92 (13.5)	35 (21.1 %)	16 (34.0 %)	67 (100.0)	108 (100.0)
1–9 %	45 (6.6)	22 (13.3 %)	8 (17.0 %)		
≥10 %	540 (79.2)	109 (65.7 %)	22 (46.8 %)		
Not specified	5 (0.7)		1 (2.1 %)		
KI-67					
<14 %	611 (89.6)		13 (27.7 %)	12 (17.9)	25 (23.1)
14–19 %	71 (10.4)	24 (14.5 %)	12 (25.5 %)	15 (22.4)	4 (3.7)
≥20 %		142 (85.5 %)	22 (46.8 %)	40 (59.7)	78 (72.2)
Not specified					1 (0.9)
HER2					
Negative	682 (100.0)	166 (100.0 %)			108 (100.0)
Positive			47 (100.0 %)	67 (100.0)	
CK56/EGFR/VIM (Basal phenotype)					
Negative	615 (90.2)	146 (88.0 %)	38 (80.9 %)	23 (34.3)	18 (16.7)
Positive	48 (7.0)	15 (9.0 %)	8 (17.0 %)	42 (62.7)	88 (81.5)
Not specified.	19 (2.8)	5 (3.0 %)	1 (2.1 %)	2 (3.0)	2 (1.9)
ALDH1					
Negative	668 (97.9)	160 (96.4 %)	43 (91.5 %)	55 (82.1)	94 (87.0)
Positive	10 (1.5)	6 (3.6 %)	4 (8.5 %)	11 (16.4)	14 (13.0)
Not specified	4 (0.6)			1 (1.5)	
CD24					
Negative	452 (66.3)	86 (51.8 %)	20 (42.6 %)	31 (46.3)	69 (63.9)
Positive	225 (33.0)	80 (48.2 %)	27 (57.4 %)	36 (53.7)	39 (36.1)
Not specified	5 (0.7)				
CD44					
Negative	300 (44.0)	85 (51.2 %)	34 (72.3 %)	35 (52.2)	36 (33.3)
Positive	364 (53.4)	80 (48.2 %)	13 (27.7 %)	31 (46.3)	69 (63.9)
Not specified	18 (2.6)	1 (0.6 %)		1 (1.5)	3 (2.8)
CD24 and CD44 (Claudin phenotype)					
CD24−/CD44+	236 (34.6)	45 (27.1 %)	4 (8.5 %)	13 (19.4)	44 (40.7)
CD24+/CD44−	95 (13.9)	45 (27.1 %)	18 (38.3 %)	18 (26.9)	14 (13.0)
Other associations	331 (48.5)	75 (45.2 %)	25 (53.2 %)	35 (52.2)	47 (43.5)
Not specified	20 (2.9)	1 (0.6 %)		1 (1.5)	3 (2.8)
E-Cadherine					
Negative	86 (12.6)	2 (1.2 %)	4 (8.5 %)	3 (4.5)	7 (6.5)
Positive	590 (86.5)	164 (98.8 %)	43 (91.5 %)	63 (94.0)	99 (91.7)
Not specified	6 (0.9)			1 (1.5)	2 (1.9)
TRIO phenotype					
Negative	315 (46.2)	85 (51.2 %)	22 (46.8 %)	31 (46.3)	59 (54.6)
Positive	352 (51.6)	81 (48.8 %)	24 (51.1 %)	36 (53.7)	48 (44.4)
Not specified	15 (2.2)		1 (2.1 %)		1 (0.9)
BCL2 phenotype					
Negative	166 (24.3)	58 (34.9 %)	27 (57.4 %)	61 (91.0)	95 (88.0)
Positive	506 (74.2)	106 (63.9 %)	20 (42.6 %)	6 (9.0)	11 (10.2)
Not specified	10 (1.5)	2 (1.2 %)			2 (1.9)

Comparisons between basal-like and non-basal-like phenotypes in triple negative tumors (Table 2) showed that basal tumors were more often proliferative (Ki67 > 15 %) (*P* < 0.001), mSBR grade 3 (<0.001), with extensive necrosis (*P* = 0.007), marked inflammation (*P* = 0.07), CD44+ (70.5 % versus 37 %; *P* = 0.008), CD24−/CD44+ (*P* = 0.046), ALDH1 and Trio positive (*P* = 0.11 and *P* = 0.008, respectively).

**Table 2:** Immunohistochemical parameters for basal-like and non-basal-like triple negative tumors

	Non basal-like (n = 16)	Basal-like (n = 88)
KI67		
<14 %	12 (75.0)	12 (13.6)
14–19 %		4 (4.5)
≥20 %	4 (25.0)	72 (81.8)
CK5-6/EGFR/VIM (Basal phenotype)		
Negative	16 (100.0)	
Positive		88 (100.0)
ALDH1		
Negative	16 (100.0)	74 (84.1)
Positive		14 (15.9)
Cd24_pos		
Negative	8 (50.0)	57 (64.8)
Positive	8 (50.0)	31 (35.2)
CD44		
Negative	10 (62.5)	25 (28.4)
Positive	6 (37.5)	62 (70.5)
Not specified		1 (1.1)
CD24 and CD44 (Claudin phenotype)		
CD24−/CD44+	3 (18.8)	40 (45.5)
CD24+/CD44−	5 (31.3)	9 (10.2)
Other associations	8 (50.0)	38 (43.2)
Not specified		1 (1.1)
E-Cadherine		
Negative	5 (31.3)	1 (1.1)
Positive	11 (68.8)	87 (98.9)
TRIO phenotype		
Negative	4 (25.0)	53 (60.2)
Positive	12 (75.0)	34 (38.6)
Not specified		1 (1.1)
BCL2 phenotype		
Negative	13 (81.3)	79 (89.8)
Positive	2 (12.5)	8 (9.1)
Not specified	1 (6.3)	1 (1.1)

### Distant metastasis-free survival at 5 years

The 284 distant metastases (26 %) are presented in Table 3. LBH+ patients had the highest metastatic rate, followed by H2+, TN and LBH− patients. Metastatic events were the least frequent in the LA group of patients. H2+ and TN (42 and 31 % respectively) appear to be associated with higher rates of cerebral metastases compared to LA and LBH− and LBH+ molecular groups (6, 15 and 20 % respectively).

**Table 3:** Metastatic events at 5 years according to molecular groups

	LA (n = 682) (%)	LBH− (n = 166) (%)	LBH+ (n = 47) (%)	H2+ (n = 67) (%)	TN (n = 108) (%)
Metastases	149 (21.8)	55 (33.1)	20 (42.6)	24 (35.8)	36 (33.3)
Bone	102 (68.4)	38 (69.1)	16 (80)	13 (54.2)	17 (47.3)
Lung	39 (26.7)	26 (47.3)	5 (25)	10 (41.7)	13 (36.1)
Liver	58 (38.9)	25 (45.5)	10 (50)	12 (50.0)	11 (30.6)
Brain	9 (6.0)	8 (14.6)	4 (20)	10 (41.7)	11 (30.6)

**Fig. 1:**
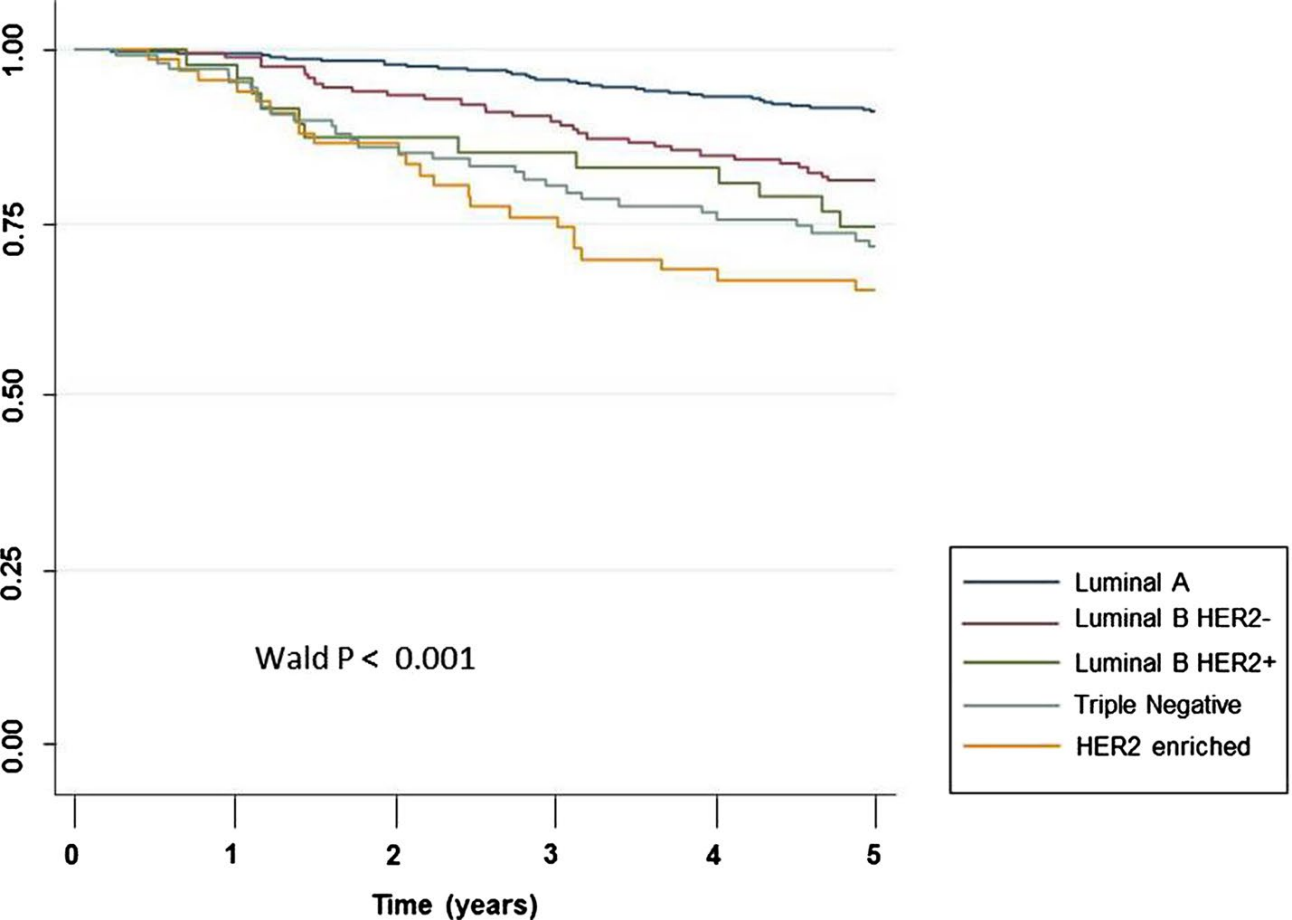
Distant metastasis-free survival over the first 5 years across five breast cancer molecular groups (n = 1070)

DMFS in LA (90 %) was better than in LBH− (80.9 %), hazard ratio (HR) = 2.22 [1.44–3.43] *P* < 0.001; LBH+ (74.5 %), HR = 3.14 [1.69–5.84] *P* < 0.001, TN (71.5 %) HR = 3.63 [2.34–5.63], *P* < 0.001; and H2+ (65.2 %), HR = 4.69 [2.90–7.59], *P* < 0.001 (Fig. 1). DMFS in LBH− was better than in H2+, HR = 2.1 [1.22–3.61] *P* = 0.007 and not different from LBH+, HR = 1.41 [0.72–2.75] *P* = 0.3 nor TN, HR = 1.63 [0.98–2.69] *P* = 0.056. Survival was not different between LBH+ and H2+ patients, HR = 1.49 [0.74–2.99] *P* = 0.26 and between TN and H2+ patients, HR = 1.29 [0.75–2.22] *P* = 0.35.

**Fig. 2:**
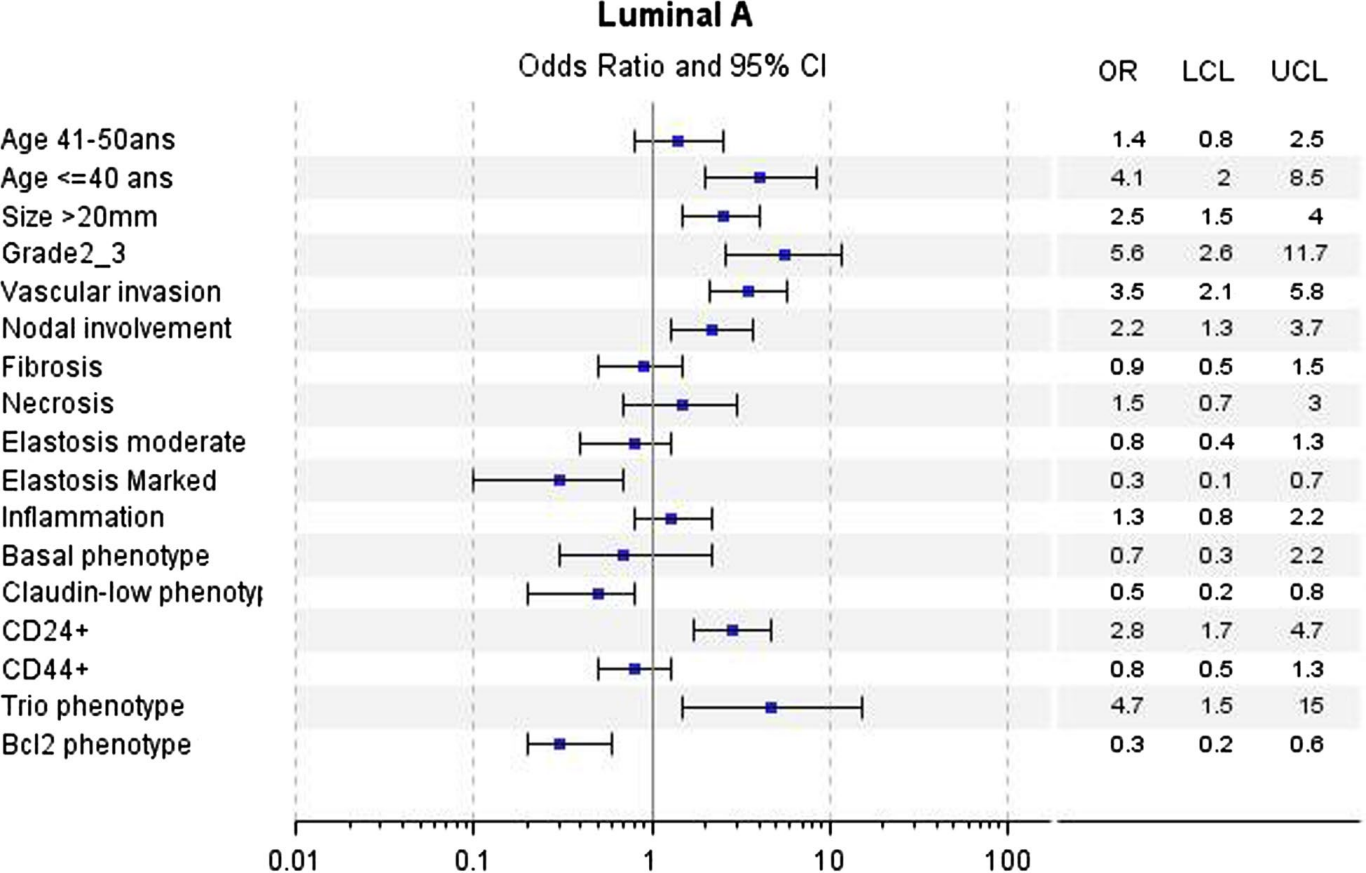
Association between clinicopathological features, immunohistochemical factors and distant metastasis-free survival over the first 5 years in the Luminal A group of breast cancer patients

**Fig. 3:**
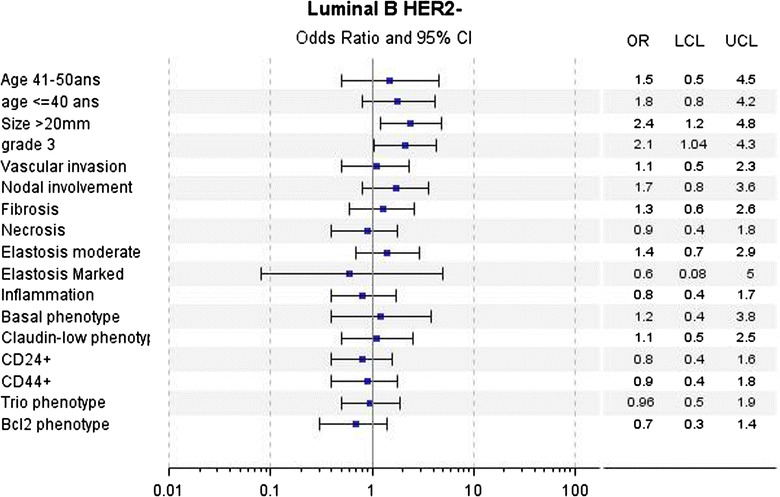
Association between clinicopathological features, immunohistochemical factors and distant metastasis-free survival over the first 5 years in the Luminal B Her2-negative group of breast cancer patients

**Fig. 4:**
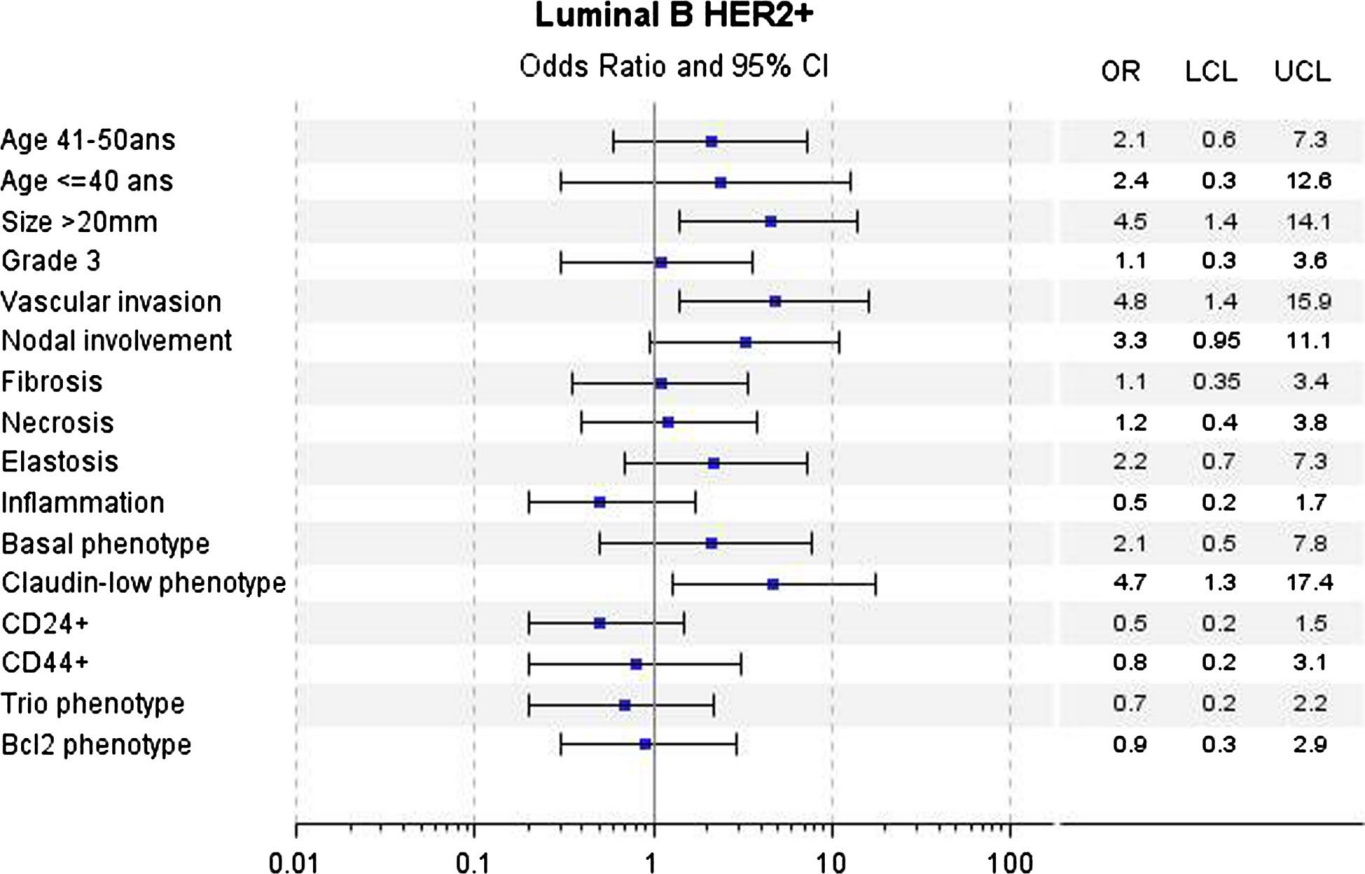
Association between clinicopathological features, immunohistochemical factors and distant metastasis-free survival over the first 5 years in the Luminal B Her2-positive group of breast cancer patients

**Fig. 5:**
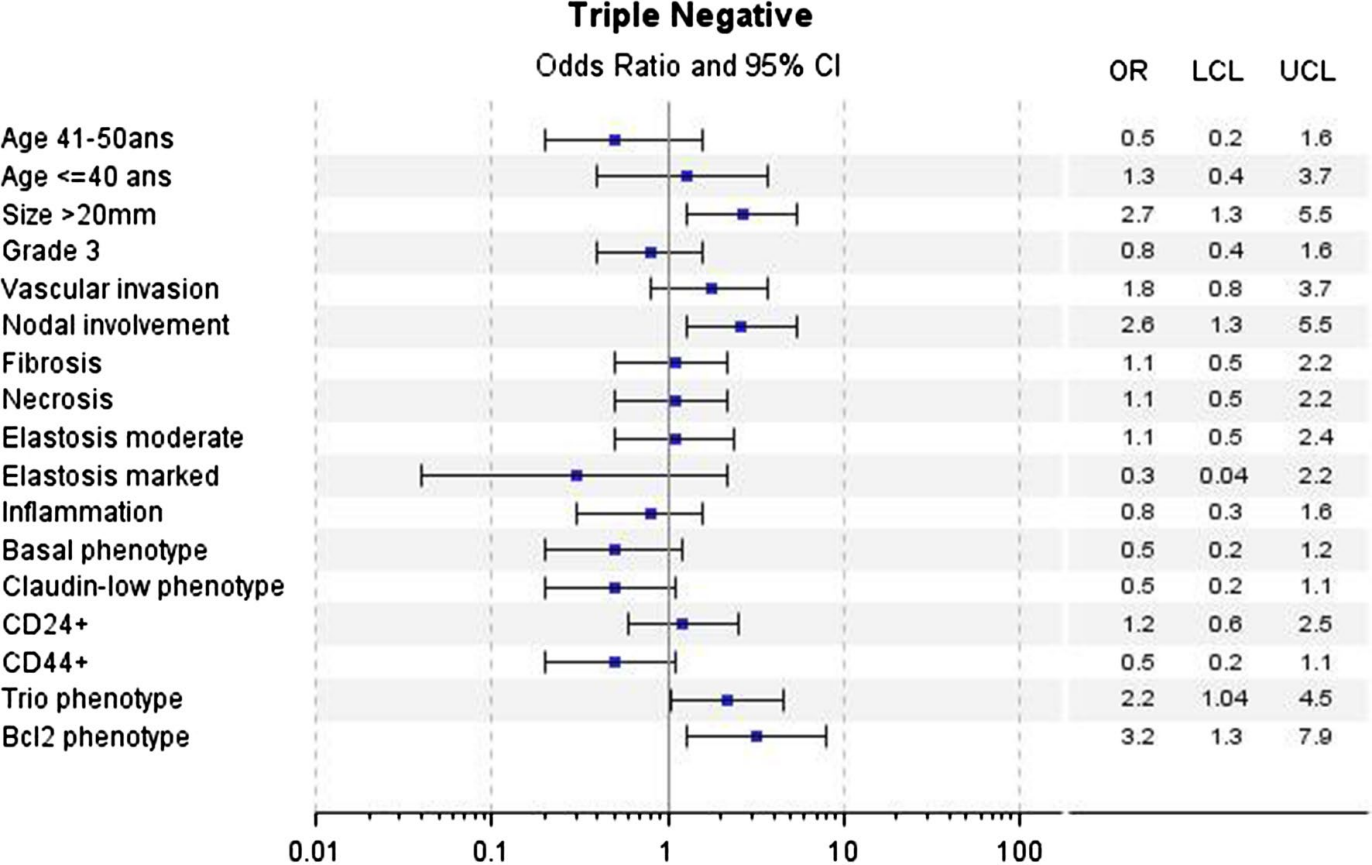
Association between clinicopathological features, immunohistochemical factors and distant metastasis-free survival over the first 5 years in the Triple negative group of breast cancer patients

**Fig. 6:**
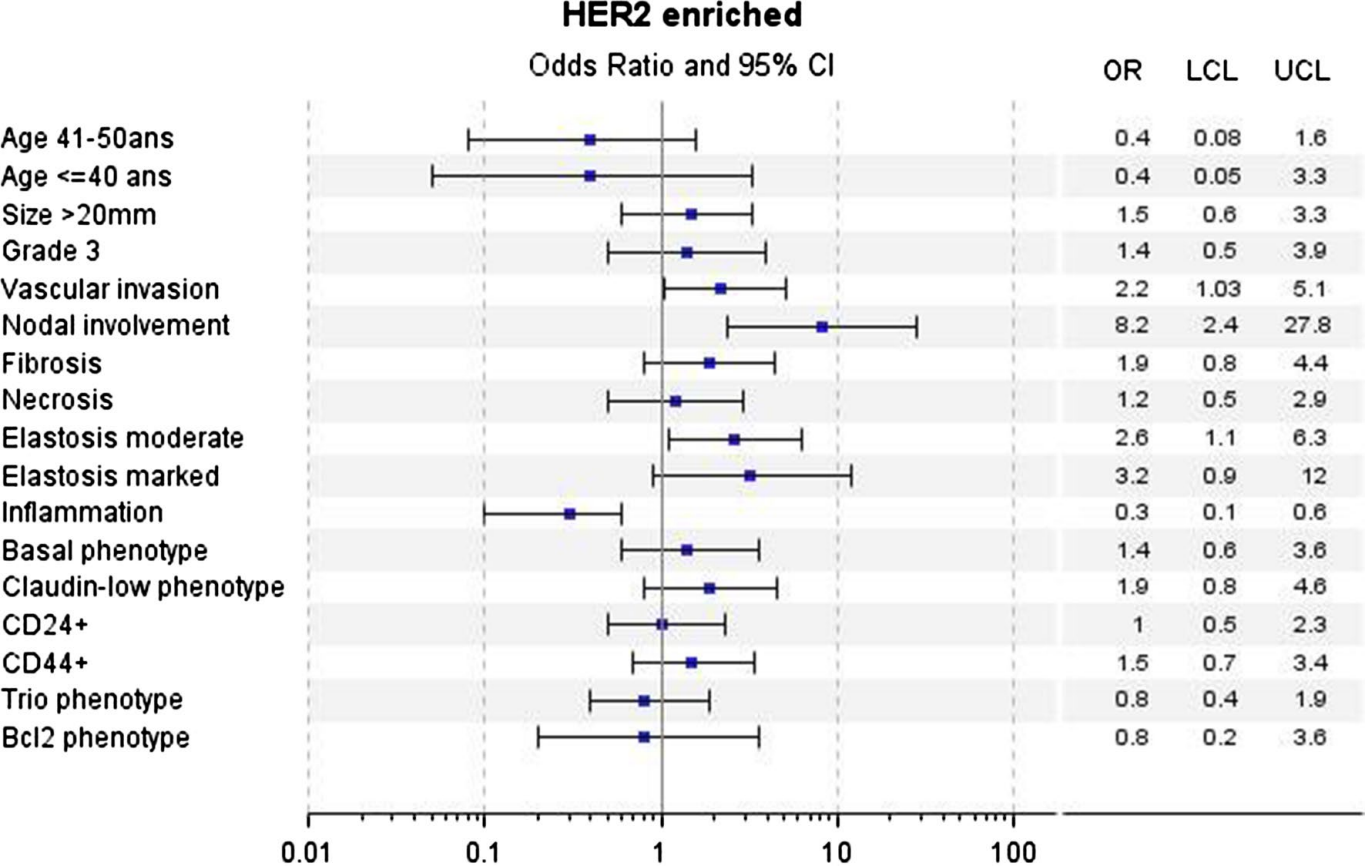
Association between clinicopathological features, immunohistochemical factors and distant metastasis-free survival over the first 5 years in the Her2-enriched group of breast cancer patients

In univariate analyses for LA tumors, a poorer DMFS over the first 5 years was associated with young age (≤40 years), large tumor size (20 mm), higher mSBR (grade 2 + 3 versus 1), vascular invasion, axillary nodal involvement, CD24 and Trio phenotypes. Abundant elastosis, CD24−/CD44+ (Claudin-low phenotype) and BCL2 phenotypes were associated with better DMFS (Fig. 2). For LBH− tumors, large tumor size (20 mm), high SBR grade (grade 3 versus 1 + 2) were associated with poorer DMFS (Fig. 3). For LBH+ tumors, large tumor size (20 mm), vascular invasion and Claudin-low phenotype were associated with poorer DMFS (Fig. 4). For TN tumors, a poorer DMFS was associated with larger tumor size (>20 mm), axillary nodal involvement, Trio and BCL2 phenotypes (Fig. 5). In H2+ tumors, axillary nodal involvement was associated with poorer DMFS, while presence of inflammation was associated with better DMFS (Fig. 6). The basal phenotype in the main molecular groups had no prognostic impact.

Multivariate models were constructed including clinical and morphological immunophenotypes significant in the univariate analyses in each molecular group (Table 3). For LA tumors, poorer DMFS was associated with younger age (≤40 years) [HR = 3.6 (1.7–7.5), *P* = 0.004], higher mSBR grade (2 or 3) [HR = 3.2 (1.5–6.9), *P* = 0.003], vascular invasion [HR = 2.3 (1.4–3.9), *P* = 0.002], CD24 expression [HR = 2.3 (1.3–3.8), *P* = 0.002], Trio expression [HR = 3.9 (1.2–12.3), *P* = 0.02], and absence of the BCL2 phenotype [HR = 2.5 (1.5–4.1), *P* < 0.001]. For LBH− tumors, poorer DMFS was associated with larger tumoral size (>20 mm) [HR = 2.4 (1.2–4.8), *P* = 0.02]. For LBH+ tumors poorer DMFS was associated with tumor size (>20 mm) [HR = 4.2 (1.3–13.7), *P* = 0.02], vascular invasion [HR = 5.7 (1.6–20.9), *P* = 0.008] and Claudin-low phenotype [HR = 6.6 (1.6–27.2), *P* = 0.009]. For TN tumors, larger tumor size [HR = 2.5 (1.2–5.3), *P* = 0.02], nodal involvement [HR = 3.5 (1.6–7.6), *P* = 0.002], Trio [HR = 2.4 (1.1–5.1), *P* = 0.03], and BCL2 [HR = 3.3 (1.3–8.4), *P* = 0.01] phenotypes were associated with poorer DMFS. In H2+ tumors, nodal involvement [HR = 8.0 (2.4–27.1), *P* = 0.01] and absence or moderate inflammation [HR = 3.7 (1.6–8.5), *P* = 0.004] were associated with poorer DMFS.

### Distant metastasis-free survival at 10 years

**Fig. 7:**
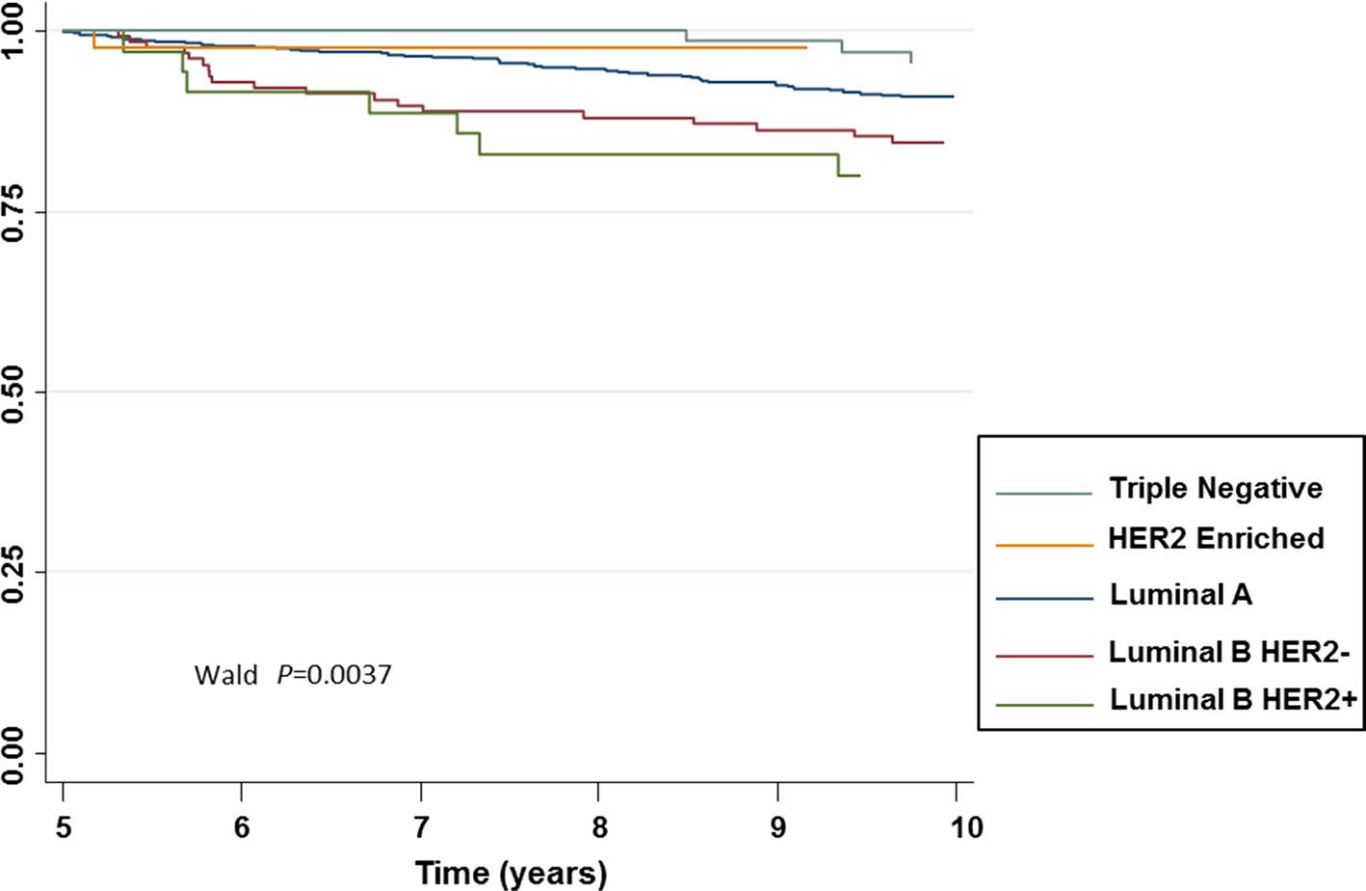
Distant metastasis-free survival after 5 years for all patients alive and with no events at 5 years across five breast cancer molecular groups (n = 883)

For patients alive with no distant metastatic events at 5 years, the 10-year DMFS was similar in LA (90.8 %) and LB (83.4 %) patients, HR = 1.4 [0.9–2.1], *P* = 0.06 (Fig. 7). There were too few events after 5 years in the LBH+ (n = 8), TN (n = 6) and H2+ (n = 1) subgroups to enable analyses. Therefore, data on LBH− and LBH+ patients were analyzed in one group called LB.

A poorer DMFS after first 5 years in LA tumors was associated with large tumor size (20 mm), and nodal involvement (Additional file 3: Table S2a). For LB tumors, poorer DMFS was associated with young age (≤40 years), nodal involvement and basal phenotype. In multivariate analyses, nodal involvement [HR = 2.1 (1.4–3.4) *P* = 0.001] as well as tumor size [HR = 1.8 (1.1–3.0), *P* = 0.02] remained significant prognostic factors for poorer DMFS in the LA group of patients. Young age was the sole independent pejorative factor in the LB group [HR = 5.4 (2.4–12.0), *P* < 0.001].

## Discussion

In this study, we show that clinical and morphological features as well as survival characteristics of breast cancer patients vary significantly across the different molecular groups.

Overall, DMFS over the first 5 years was better in LA than in all other groups. There were no DMFS differences between LA and LB tumors after the first 5 years, although it has been reported that Luminal tumors have a better 5-year survival than non-Luminal (Blows et al. 2010) and that HER2+-enriched tumors have the worst survival of all groups (Cheang et al. 2008). None of the ER-negative phenotypes in our study were associated with DMFS over 10 years for patients with no events at 5 years.

In contrast to other reports (Tischkowitz et al. 2007), Cheang et al. reported poorer survival in TN tumors with *basal phenotypes* compared to TN tumors without (Cheang et al. 2008). Similarly, the basal phenotype (CK5/6 and or EGFR positive) was associated with shorter DMFS in luminal HER2-negative and TN but not in the HER2-enriched subgroups (Blows et al. 2010). In our results, the basal phenotype was not associated with poorer survival, most likely due to the smaller series size than in previous reports.

We tested *Trio* for the first time in a large series of breast cancer and it was independently associated with poorer survival in LA and TN tumors. This complex protein activates the Rho-GTPases, Rac1 and RhoA, by its two guanine nucleotide exchange factor (GEF) domains (Debant et al. 1996). Cells expressing the Racl-specific amino-terminal guanine nucleotide exchange factor domain display more rapid cell spreading, haptotactic cell migration and anchorage-independent growth suggesting that Trio regulates both cell motility and cell growth (Seipel et al. 1999). Expression of Tgat, an oncogenic isoform of Trio, induces a strong RhoA activation and the formation of tumors in a xenograft mouse model (Bouquier et al. 2009). Furthermore, breast cancer patients with poor prognosis exhibit high levels of Trio (Lane et al. 2008).

CD24−/CD44+ tumor cells or ALDH1-positive tumor cells were significantly associated with poor survival in a recent meta-analysis (Zhou et al. 2010), although ALDH1 expression alone does not significantly predict outcomes (Neumeister et al. 2010). In our study, the Claudin-low phenotype was an independent factor for shorter DMFS in the Luminal B HER2-positive group of patients only. Elsewhere, the CD24−/CD44+ phenotype was conversely associated with a better survival (as in LA, by univariate analysis only, in our study). CD24 expression was a marker of poor prognosis in LA in our study. CD44-positive cells represent progenitor-like cells and CD24 positive cells represent more differentiated luminal epithelial cells (Campbell and Polyak 2007; Honeth et al. 2008). CD24 plays a role in facilitating metastasis by the interaction between tumor cells and platelets or endothelial cells and is also associated with proliferation, adhesion and invasion in MCF-7 breast cancer cells (Kim et al. 2011) affecting their CXCR4 function (Schabath et al. 2006).

In the literature, BCL2 is reported as a favorable prognostic factor in ER-positive breast cancers, that is independent of the Nottingham Prognostic index (Callagy et al. 2008) and adjuvant therapy received (Dawson et al. 2010). In our study, the expression of BCL2 was an independent favorable factor in LA, and an unfavorable prognostic factor in TN. Recently the adverse prognosis associated with BCL2 expression in triple negative tumors has been recognized (Abdel-Fatah et al. 2013). Furthermore, BCL2 expression in associated with poor response to Anthracycline-based chemotherapy in Triple negative breast cancers (Bouchalova et al. 2015).

This study presents a few limitations. Firstly, the immunohistochemical results are interpreted on TMA in cases where the percentages are low and positivity is not always conclusive. Secondly, there have been some changes in standard treatment offered for breast cancer since the beginning of this study. For example, almost two-thirds of our patients did not receive any adjuvant treatment which is unlikely to be the case in a more recent cohort.

We have assessed the prognostic significance of CD24 and CD44, detailed the prognostic value of BCL2 in LA and TN, and highlighted the association of Trio and shorter survival in TN. Furthermore, multivariate analyses including clinical, tumoral, micro-environmental and immunohistochemical criteria revealed relevant negative or positive factors in each molecular group. The strength of these factors is emphasized in our study as 59 % of ER/PR-positive patients and 65 % of ER/PR-negative patients had no adjuvant therapy. Further work is now needed to ascertain how to apply CD24, CD44, BCL2 and Trio in relevant molecular groups to define new clinicophenotypic models and to identify new therapeutic strategies.

## Additional files

Additional file 1: Annex A (online only) – *Tissue micro array and Immunohistochemical assay*

Additional file 2: Table S1a–e. Comparison of clinicopathologic features between LA and LBH− groups.
Additional file 3: Table S2a. Univariate analyses for associations between clinicopathological, microenviromental and immunohistochemical phenotypes and distant metastasis-free survival (DMFS) after five years according to luminal A and luminal B groups* (for patients with no events at 5 years).
