# Supplementary material for: Comprehensive prognostic analysis in breast cancer integrating clinical, tumoral, micro-environmental and immunohistochemical criteria
Source: Springerplus. 2015 Sep 21;4:528. doi: 10.1186/s40064-015-1297-8 (PMC4576021; doi:10.1186/s40064-015-1297-8)
Supplement: Supplementary file 1 — Additional file 1: Annex A (online only) – Tissue micro array and Immunohistochemical assay [file 40064_2015_1297_MOESM1_ESM.doc]

## Annex A (online only) – Tissue micro array and Immunohistochemical assay

Immunohistochemistry was performed concurrently on serial sections. Slides were treated on Benchmark®XT (Ventana). No markers other than those reported below were performed.

Dilution ratio and optimal retrieval buffer varied for each antibody: estrogen receptor (ER) (Ventana ready to use, Rabbit monoclonal, clone sp1)-CC1 standard/32 mn; Progesterone receptor (PR) (Ventana ready to use, Rabbit monoclonal, clone 1E2)-CC1 standard/32 mn; Androgen receptor (AR) (Dako, Mouse monoclonal, clone AR441) at 1/50, CC1 extended/36 min + Ampli; HER2 (Neomarkers, Rabbit monoclonal, clone SP3) at 1/100, CC2 mild/48 mn at room temperature; Ki67 (Neomarkers, Rabbit monoclonal, clone Sp6) at 1/100, CC1 standard/32 mn; Cytokeratine 5/6 (CK5/6) (Dako, Mouse monoclonal, clone D5/16B4) at 1/50, CC1 mild / 32 min; E-Cadherin (Invitrogen, Mouse monoclonal, clone 4A2C7) at 1/50, CC1 mild / 32mn; Vimentin (Dako, Mouse monoclonal, clone V9) at 1/200, CC1 mild / 20 min; Epidermal growth factor receptor (EGFR) (Ventana ready to use, clone 3C6)-Protease1 (8mn)/32mn; ALDH1 (BD Transduction Laboratories, Mouse monoclonal, clone 44/ALDH) at 1/200, CC1 standard/32 mn; BCL2 Oncoprotein (Dako, Mouse monoclonal clone 124 at 1/20 CC1 standard/32 mn+ Ampli). Mouse monoclonal anti-human CD24 (clone SN3b; Thermoscientific) and mouse monoclonal anti-human CD44 (clone 156-3C11; Labvision/Thermoscientific) were applied at respectively 1/100 and 1/200 dilution during 1 hour. Rabbit polyclonal anti-Trio antibody (Trio-MTP) was applied at 0.3mg/ml (1/100-HIER pH9) over night at 4°C. For CD24, CD44 and Trio, slides were deparaffinised and pretreated (with citrate buffer Dako pH6 for CD24 and CD44, with target retrieval solution Dako pH9 for Trio), and heat-mediated antigen retrieval solution in a microwave (Whirlpool 6th sens-JT356) at 98°C for 20 mn. For CD24 and CD44, further steps were done at room temperature. Endogenous peroxidase activity was quenched with peroxidase Block (Dako, Carpinteria) for 5 mn. After treatment with the primary antibodies, the slides were washed in TBST (Dako, wash buffer) and detected with horseradish peroxidase-conjugated anti-mouse/rabbit Envision+ kit (Dako). All slides were counterstained with haematoxylin.
