# Supplementary material for: Comprehensive prognostic analysis in breast cancer integrating clinical, tumoral, micro-environmental and immunohistochemical criteria
Source: Springerplus. 2015 Sep 21;4:528. doi: 10.1186/s40064-015-1297-8 (PMC4576021; doi:10.1186/s40064-015-1297-8)
Supplement: Supplementary file 2 — Additional file 2: Table S1a–e. Comparison of clinicopathologic features between LA and LBH− groups. [file 40064_2015_1297_MOESM2_ESM.docx]

**Supplementary Tables**

**Supplementary Table S1a. Comparison of clinicopathologic features between LA and LBH- groups**

|  |  |  |  |
| --- | --- | --- | --- |
|  | Luminal A (n=682) (%) | Chi^2^ test *P* value | Luminal B HER2 negative (n=166) (%) |
|  |  |  |  |
| Age |  |  |  |
| <=40y | 34 (5.0) |  | 17 (10.2%) |
| 41-50 y | 151 (22.1) | 0.009 | 25 (15.1%) |
| >50y | 497 (72.9) |  | 124 (74.7%) |
| Histological size |  |  |  |
| <20mm | 561 (82.3) |  | 112 (67.5%) |
| >=20mm | 105 (15.4) | <0.001 | 52 (31.3%) |
| Not specified | 16 (2.3) |  | 2 (1.2%) |
| Mitoses per10Hpf |  |  |  |
| <=5 | 383 (56.2) |  | 13 (7.8%) |
| juin-15 | 230 (33.7) | <0.001 | 69 (41.6%) |
| >15 | 57 (8.4) |  | 84 (50.6%) |
| Not specified | 12 (1.8) |  |  |
| mSBR grade |  |  |  |
| 1 | 298 (43.7) |  | 7 (4.2%) |
| 2 | 327 (47.9) | <0.001 | 96 (57.8%) |
| 3 | 53 (7.8) |  | 63 (38.0%) |
| Not specified | 4 (0.6) |  |  |
| Vascular invasion |  |  |  |
| absent | 533 (78.2) | <0.001 | 99 (59.6%) |
| present | 142 (20.8) |  | 67 (40.4%) |
| Not specified | 7 (1.0) |  |  |
| Nodal status |  |  |  |
| N- | 403 (59.1) |  | 71 (42.8%) |
| N+ | 261 (38.3) | <0.001 | 92 (55.4%) |
| Not specified | 18 (2.6) |  | 3 (1.8%) |
| Inflammation |  |  |  |
| absent | 439 (64.4) | Absence vs Presence | 89 (53.6%) |
| Moderate | 229 (33.6) | 0.009 | 68 (41.0%) |
| Marked | 8 (1.2) |  | 8 (4.8%) |
| Not specified | 6 (0.9) |  | 1 (0.6%) |
| Fibrosis |  |  |  |
| absent | 12 (1.8) | 0.037 | 7 (4.2%) |
| Moderate | 290 (42.5) |  | 81 (48.8%) |
| Marked | 374 (54.8) |  | 77 (46.4%) |
| Not specified | 6 (0.9) |  | 1 (0.6%) |
| Elastosis |  |  |  |
| absent | 185 (27.1) |  | 69 (41.6%) |
| Moderate | 339 (49.7) | <0.001 | 86 (51.8%) |
| Marked | 152 (22.3) |  | 10 (6.0%) |
| Not specified | 6 (0.9) |  | 1 (0.6%) |
| Necrosis |  |  |  |
| absent | 604 (88.6) | Absence vs Presence | 106 (63.9%) |
| Moderate | 69 (10.1) |  | 54 (32.5%) |
| Marked | 3 (0.4) | <0.0010 | 5 (3.0%) |
| Not specified | 6 (0.9) |  | 1 (0.6%) |
| Treatment** |  |  |  |
| No treatment | 424 (62.2) |  | 77 (46.4%) |
| Tamoxifen | 177 (26.0) |  | 58 (34.9%) |
| Chemo, | 68 (10.0) |  | 28 (16.9%) |
| Tam.+Chemo | 13 (1.9) |  | 3 (1.8%) |

**Supplementary Table S1b. Comparison of clinicopathologic features between LA and LBH+ groups**

|  |  |  |  |
| --- | --- | --- | --- |
|  | Luminal A (n=682) (%) | Chi^2^ test *P* value | Luminal B HER2 positive (n=47) (%) |
|  |  |  |  |
| Age |  |  |  |
| <=40y | 34 (5.0) |  | 3 (6.4%) |
| 41-50 y | 151 (22.1) | 0.0014 | 21 (44.7%) |
| >50y | 497 (72.9) |  | 23 (48.9%) |
| Histological size |  |  |  |
| <20mm | 561 (82.3) | 0.034 | 34 (72.3%) |
| >=20mm | 105 (15.4) |  | 13 (27.7%) |
| Not specified | 16 (2.3) |  |  |
| Mitoses per 10Hpf |  |  |  |
| <=5 | 383 (56.2) |  | 5 (10.6%) |
| juin-15 | 230 (33.7) | <0.001 | 26 (55.3%) |
| >15 | 57 (8.4) |  | 15 (31.9%) |
| Not specified | 12 (1.8) |  | 1 (2.1%) |
| mSBR grade |  |  |  |
| 1 | 298 (43.7) | 1 and 2 vs 3 | 4 (8.5%) |
| 2 | 327 (47.9) | <0.001 | 27 (57.4%) |
| 3 | 53 (7.8) |  | 16 (34.0%) |
| Not specified | 4 (0.6) |  |  |
| Vascular invasion |  |  |  |
| absent | 533 (78.2) | 0.015 | 30 (63.8%) |
| present | 142 (20.8) |  | 17 (36.2%) |
| Not specified | 7 (1.0) |  |  |
| Nodal status |  |  |  |
| N- | 403 (59.1) |  | 27 (57.4%) |
| N+ | 261 (38.3) | 0.92 | 18 (38.3%) |
| Not specified | 18 (2.6) |  | 2 (4.3%) |
| Inflammation |  |  |  |
| absent | 439 (64.4) | 0.015 | 20 (42.6%) |
| Moderate | 229 (33.6) |  | 23 (48.9%) |
| Marked | 8 (1.2) |  | 4 (8.5%) |
| Not specified | 6 (0.9) |  |  |
| Fibrosis |  |  |  |
| absent | 12 (1.8) |  | 3 (6.4%) |
| Moderate | 290 (42.5) | 0.09 | 21 (44.7%) |
| Marked | 374 (54.8) |  | 23 (48.9%) |
| Not specified | 6 (0.9) |  |  |
| Elastosis |  |  |  |
| absent | 185 (27.1) | Absence vs Presence | 24 (51.1%) |
| Moderate | 339 (49.7) | <0.001 | 23 (48.9%) |
| Marked | 152 (22.3) |  |  |
| Not specified | 6 (0.9) |  |  |
| Necrosis |  |  |  |
| absent | 604 (88.6) | Absence vs Presence | 28 (59.6%) |
| Moderate | 69 (10.1) |  | 0 |
| Marked | 3 (0.4) | <0.001 | 19 (40.4%) |
| Not specified | 6 (0.9) |  |  |
| Treatment |  |  |  |
| No treatment | 424 (62.2) |  | 27 (57.4%) |
| Tamoxifen | 177 (26.0) |  | 9 (19.1%) |
| Chemo, | 68 (10.0) |  | 11 (23.4%) |
| Tam.+Chemo | 13 (1.9) |  |  |

**Supplementary Table S1c. Comparison of clinicopathologic features between LBH- and LBH+ groups**

|  | Luminal B HER2neg (n=166)(%) | Chi^2^ test *P* value | Luminal B HER2 pos (n=47) (%) |
| --- | --- | --- | --- |
|  |  |  |  |
| Age |  |  |  |
| <=40y | 17 (10.2%) |  | 3 (6.4%) |
| 41-50 y | 25 (15.1%) | <0.001 | 21 (44.7%) |
| >50y | 124 (74.7%) |  | 23 (48.9%) |
| Histological size |  |  |  |
| <20mm | 112 (67.5%) |  | 34 (72.3%) |
| >=20mm | 52 (31.3%) | 0.60 | 13 (27.7%) |
| Not specified | 2 (1.2%) |  |  |
| Mitoses per 10Hpf |  |  |  |
| <=5 | 13 (7.8%) |  | 5 (10.6%) |
| juin-15 | 69 (41.6%) | 0.090 | 26 (55.3%) |
| >15 | 84 (50.6%) |  | 15 (31.9%) |
| Not specified |  |  | 1 (2.1%) |
| mSBR grade |  |  |  |
| 1 | 7 (4.2%) | 1 and 2 vs 3 | 4 (8.5%) |
| 2 | 96 (57.8%) | 0.62 | 27 (57.4%) |
| 3 | 63 (38.0%) |  | 16 (34.0%) |
| Not specified |  |  |  |
| Vascular invasion |  |  |  |
| absent | 99 (59.6%) | 0.60 | 30 (63.8%) |
| present | 67 (40.4%) |  | 17 (36.2%) |
| Not specified |  |  |  |
| Nodal status |  |  |  |
| N- | 71 (42.8%) | 0.05 | 27 (57.4%) |
| N+ | 92 (55.4%) |  | 18 (38.3%) |
| Not specified | 3 (1.8%) |  | 2 (4.3%) |
| Inflammation |  |  |  |
| absent | 89 (53.6%) | Absence vs Presence | 20 (42.6%) |
| Moderate | 68 (41.0%) | 0.17 | 23 (48.9%) |
| Marked | 8 (4.8%) |  | 4 (8.5%) |
| Not specified | 1 (0.6%) |  |  |
| Fibrosis |  |  |  |
| absent | 7 (4.2%) |  | 3 (6.4%) |
| Moderate | 81 (48.8%) | Absence vs Presence | 21 (44.7%) |
| Marked | 77 (46.4%) | 0.69 | 23 (48.9%) |
| Not specified | 1 (0.6%) |  |  |
| Elastosis |  |  |  |
| absent | 69 (41.6%) |  | 24 (51.1%) |
| Moderate | 86 (51.8%) | Marked vs oter | 23 (48.9%) |
| Marked | 10 (6.0%) | 0.26 |  |
| Not specified | 1 (0.6%) |  |  |
| Necrosis |  |  |  |
| absent | 106 (63.9%) | Absence vs Presence | 28 (59.6%) |
| Moderate | 54 (32.5%) | 0.56 | 0 |
| Marked | 5 (3.0%) |  | 19 (40.4%) |
| Not specified | 1 (0.6%) |  |  |
| Treatment |  |  |  |
| No treatment | 77 (46.4%) |  | 27 (57.4%) |
| Tamoxifen | 58 (34.9%) |  | 9 (19.1%) |
| Chemo, | 28 (16.9%) |  | 11 (23.4%) |
| Tam.+Chemo | 3 (1.8%) |  |  |

**Supplementary Table S1d. Comparison of clinicopathologic features between LBH+ and H2+ groups**

|  | Luminal B HER2pos (n=47) (%) | Chi^2^ test *P* value | HER-2 enriched (n=67) (%) |
| --- | --- | --- | --- |
|  |  |  |  |
| Age |  |  |  |
| <=40y | 3 (6.4%) |  | 5 (7.5) |
| 41-50 y | 21 (44.7%) | <0.001 | 11 (16.4) |
| >50y | 23 (48.9%) |  | 51 (76.1) |
| Histological size |  |  |  |
| <20mm | 34 (72.3%) | 0.39 | 42 (62.7) |
| >=20mm | 13 (27.7%) |  | 23 (34.3) |
| Not specified |  |  | 2 (3.0) |
| Mitoses per 10Hpf |  |  |  |
| <=5 | 5 (10.6%) | 1 and 2 vs 3 | 4 (6.0) |
| juin-15 | 26 (55.3%) | <0.001 | 17 (25.4) |
| >15 | 15 (31.9%) |  | 45 (67.2) |
| Not specified | 1 (2.1%) |  | 1 (1.5) |
| mSBR grade |  |  |  |
| 1 | 4 (8.5%) | 1 and 2 vs 3 | 3 (4.5) |
| 2 | 27 (57.4%) | <0.001 | 10 (14.9) |
| 3 | 16 (34.0%) |  | 53 (79.1) |
| Not specified |  |  | 1 (1.5) |
| Vascular invasion |  |  |  |
| absent | 30 (63.8%) | 0.98 | 42 (62.7) |
| present | 17 (36.2%) |  | 24 (35.8) |
| Not specified |  |  | 1 (1.5) |
| Nodal status |  |  |  |
| N- | 27 (57.4%) |  | 31 (46.3) |
| N+ | 18 (38.3%) | 0.18 | 35 (52.2) |
| Not specified | 2 (4.3%) |  | 1 (1.5) |
| Inflammation |  |  |  |
| absent | 20 (42.6%) | Absence vs Presence | 16 (23.9) |
| Moderate | 23 (48.9%) | 0.039 | 39 (58.2) |
| Marked | 4 (8.5%) |  | 11 (16.4) |
| Not specified |  |  | 1 (1.5) |
| Fibrosis |  |  |  |
| absent | 3 (6.4%) |  | 2 (3.0) |
| Moderate | 21 (44.7%) | Marked vs Other | 28 (41.8) |
| Marked | 23 (48.9%) | 0.56 | 36 (53.7) |
| Not specified |  |  | 1 (1.5) |
| Elastosis |  |  |  |
| absent | 24 (51.1%) |  | 38 (56.7) |
| Moderate | 23 (48.9%) | Absence vs Presence | 23 (34.3) |
| Marked |  | 0.49 | 5 (7.5) |
| Not specified |  |  | 1 (1.5) |
| Necrosis |  |  |  |
| absent | 28 (59.6%) |  | 26 (38.8) |
| Moderate | 0 | Marked vs Other | 36 (53.7) |
| Marked | 19 (40.4%) | <0.001 | 4 (6.0) |
| Not specified |  |  | 1 (1.5) |
| Treatment |  |  |  |
| No treatment | 27 (57.4%) |  | 44 (65.7) |
| Tamoxifen | 9 (19.1%) |  | 6 (9.0) |
| Chemo, | 11 (23.4%) |  | 14 (20.9) |
| Tam.+Chemo |  |  | 3 (4.5) |

**Supplementary Table S1e. Comparison of clinicopathologic features between H2+ and TN groups**

|  | Her-2 enriched (n=67) (%) | Chi^2^ test *P* value | Triple negative (N=108) (%) |
| --- | --- | --- | --- |
|  |  |  |  |
| Age |  |  |  |
| <=40y | 5 (7.5) |  | 12 (11.1) |
| 41-50 y | 11 (16.4) | 0.47 | 23 (21.3) |
| >50y | 51 (76.1) |  | 73 (67.6) |
| Histological size |  |  |  |
| <20mm | 42 (62.7) | 0.61 | 65 (60.2) |
| >=20mm | 23 (34.3) |  | 42 (38.9) |
| Not specified | 2 (3.0) |  | 1 (0.9) |
| Mitoses per 10 Hpf |  |  |  |
| <=5 | 4 (6.0) |  | 15 (13.9) |
| juin-15 | 17 (25.4) | 0.23 | 22 (20.4) |
| >15 | 45 (67.2) |  | 70 (64.8) |
| Not specified | 1 (1.5) |  | 1 (0.9) |
| mSBR grade |  |  |  |
| 1 | 3 (4.5) |  | 8 (7.4) |
| 2 | 10 (14.9) | 0.47 | 22 (20.4) |
| 3 | 53 (79.1) |  | 78 (72.2) |
| Not specified | 1 (1.5) |  |  |
| Vascular invasion |  |  |  |
| absent | 42 (62.7) | 0.18 | 79 (73.1) |
| present | 24 (35.8) |  | 29 (26.9) |
| Not specified | 1 (1.5) |  |  |
| Nodal status |  |  |  |
| N- | 31 (46.3) |  | 66 (61.1) |
| N+ | 35 (52.2) | 0.034 | 38 (35.2) |
| Not specified | 1 (1.5) |  | 4 (3.7) |
| Inflammation |  |  |  |
| absent | 16 (23.9) |  | 29 (26.9) |
| Moderate | 39 (58.2) | 0.70 | 57 (52.8) |
| Marked | 11 (16.4) |  | 22 (20.4) |
| Not specified | 1 (1.5) |  |  |
| Fibrosis |  |  |  |
| absent | 2 (3.0) | Marked vs Other | 8 (7.4) |
| Moderate | 28 (41.8) | 0.56 | 46 (42.6) |
| Marked | 36 (53.7) |  | 54 (50.0) |
| Not specified | 1 (1.5) |  |  |
| Elastosis |  |  |  |
| absent | 38 (56.7) |  | 64 (59.3) |
| Moderate | 23 (34.3) | Absence vs Presence | 34 (31.5) |
| Marked | 5 (7.5) | 0.83 | 10 (9.3) |
| Not specified | 1 (1.5) |  |  |
| Necrosis |  |  |  |
| absent | 26 (38.8) |  | 42 (38.9) |
| Moderate | 36 (53.7) | Marked vs Other | 48 (44.4) |
| Marked | 4 (6.0) | 0.054 | 17 (15.7) |
| Not specified | 1 (1.5) |  | 1 (0.9) |
| Treatment |  |  |  |
| No treatment | 44 (65.7) |  | 70 (64.8) |
| Tamoxifen | 6 (9.0) |  | 7 (6.5) |
| Chemo, | 14 (20.9) |  | 28 (25.9) |
| Tam.+Chemo | 3 (4.5) |  | 3 (2.8) |
