# Supplementary material for: Comprehensive prognostic analysis in breast cancer integrating clinical, tumoral, micro-environmental and immunohistochemical criteria
Source: Springerplus. 2015 Sep 21;4:528. doi: 10.1186/s40064-015-1297-8 (PMC4576021; doi:10.1186/s40064-015-1297-8)
Supplement: Supplementary file 3 — Additional file 3: Table S2a. Univariate analyses for associations between clinicopathological, microenviromental and immunohistochemical phenotypes and distant metastasis-free survival (DMFS) after five years according to luminal A and luminal B groups* (for patients with no events at 5 years). [file 40064_2015_1297_MOESM3_ESM.docx]

| **Table S2a.** Univariate analyses for associations between clinicopathological, micro-enviromental and immunohistochemical phenotypes and distant metastasis-free survival (DMFS) after five years according to Luminal A and Luminal B groups (for patients with no event at 5 years)(online only table). | | | | | |
| --- | --- | --- | --- | --- | --- |
|  | | **Luminal A** | | **Luminal B** | |
|  |  | n=605; 88 events | | n=163, 32 events | |
|  |  | HR [95%CI] | *P*-value | HR [95%CI] | *P*-value |
| Age | >50 |  | ns | 1 | <0.001 |
|  | 41-50 |  |  | 1.04 [0.4-2.8] |  |
|  | ≤40 |  |  | 5.4 [2.4-12.0] |  |
| Tumor size | ≤20 | 1 |  |  |  |
|  | >20 | 2.2 [1.3-3.6] | 0.003 |  | ns |
| Nodal involvement | N- | 1 |  | 1 |  |
|  | N+ | 1  2.4 [1.6-3.8] | <0.001 | 2.3 [1.1-4.7] | 0.03 |
| CK5-6/EGFR/Vim | No |  |  | 1 | 0.04 |
| **(Basal phenotype)** | Yes |  | ns | 2.6 [1.04-6.3] |  |
